# Nanostructured Lipid Carriers Loaded with Dexamethasone Prevent Inflammatory Responses in Primary Non-Parenchymal Liver Cells

**DOI:** 10.3390/pharmaceutics14081611

**Published:** 2022-08-02

**Authors:** Carolina Medina-Montano, Ignacio Rivero Berti, Rocío C. Gambaro, María José Limeres, Malin Svensson, Gisel Padula, Cecilia Y. Chain, José Sebastián Cisneros, Guillermo R. Castro, Stephan Grabbe, Matthias Bros, Stephan Gehring, German A. Islan, Maximiliano L. Cacicedo

**Affiliations:** 1 Department of Dermatology, University Medical Center of the Johannes Gutenberg University Mainz, Langenbeckstraße 1, 55131 Mainz, Germany; gmedinam@students.uni-mainz.de (C.M.-M.); stephan.grabbe@unimedizin-maiz.de (S.G.); mbros@uni-mainz.de (M.B.); 2 Laboratorio de Nanobiomateriales, Centro de Investigación y Desarrollo en Fermentaciones Industriales (CINDEFI), Departamento de Química, Facultad de Ciencias Exactas, Universidad Nacional de La Plata (UNLP)-CONICET (CCT La Plata), Calle 47 y 115, La Plata B1900, Argentina; iriveroberti@gmail.com; 3 Instituto de Genética Veterinaria (IGEVET, UNLP-CONICET La Plata), Facultad de Ciencias Veterinarias, Universidad Nacional de La Plata (UNLP), La Plata B1900, Argentina; rociogambaro@gmail.com (R.C.G.); gpadula@igevet.gob.ar (G.P.); 4 Children’s Hospital, University Medical Center of the Johannes-Gutenberg University Mainz, Langenbeckstr. 1, 55131 Mainz, Germany; mj.limeres@uni-mainz.de (M.J.L.); malin.svensson@uni-mainz.de (M.S.); stephan.gehring@uni-mainz.de (S.G.); 5 Instituto de Investigaciones Fisicoquímicas Teóricas y Aplicadas (CONICET-UNLP), La Plata B1900, Argentina; yamil@inifta.unlp.edu.ar (C.Y.C.); josesebastiancisneros@biol.unlp.edu.ar (J.S.C.); 6 Max Planck Laboratory for Structural Biology, Chemistry and Molecular Biophysics of Rosario (MPLbioR, UNR-MPIbpC), Partner Laboratory of the Max Planck Institute for Biophysical Chemistry (MPIbpC, MPG), Centro de Estudios Interdisciplinarios (CEI), Universidad Nacional de Rosario, Maipú 1065, Rosario S2000, Argentina; grcastro@gmail.com

**Keywords:** glucocorticoids, lipid nanoparticles, liver inflammation, liver immunology, autoimmune hepatitis, dexamethasone, drug-controlled release

## Abstract

Liver inflammation represents a major clinical problem in a wide range of pathologies. Among the strategies to prevent liver failure, dexamethasone (DXM) has been widely used to suppress inflammatory responses. The use of nanocarriers for encapsulation and sustained release of glucocorticoids to liver cells could provide a solution to prevent severe side effects associated with systemic delivery as the conventional treatment regime. Here we describe a nanostructured lipid carrier developed to efficiently encapsulate and release DXM. This nano-formulation proved to be stable over time, did not interact in vitro with plasma opsonins, and was well tolerated by primary non-parenchymal liver cells (NPCs). Released DXM preserved its pharmacological activity, as evidenced by inducing robust anti-inflammatory responses in NPCs. Taken together, nanostructured lipid carriers may constitute a reliable platform for the delivery of DXM to treat pathologies associated with chronic liver inflammation.

## 1. Introduction

Chronic liver inflammation is a characteristic of pathologies, such as chronic viral hepatitis and autoimmune hepatitis [1,2]. In these patients, persistent inflammation has been described as the main cause of hepatic fibrosis and cirrhosis [3]. Potential approaches to inhibit the fibrotic process mainly include strategies to suppress inflammation, by reducing viral load and downregulating liver NPC-driven immune response [4]. During recent decades, synthetic glucocorticoids have been widely used for the treatment of liver diseases. Although dexamethasone (DXM) possesses remarkable anti-inflammatory action, its use also leads to severe adverse effects, mainly due to immunosuppression [5]. Moreover, administration of synthetic glucocorticoids, such as DXM, to pediatric patients has been associated with severe adverse effects, like gastrointestinal perforation and decreased growth [6,7]. Multiple nanomedicine-related strategies have been studied to counteract these side effects associated with systemic delivery of soluble DXM. One of the main advantages of such approaches is the frequent observation that nanoparticles (NPs), after systemic administration, usually accumulate in the liver [8]. Furthermore, it is well established that NPs within the liver most often engage with liver non-parenchymal cells (NPCs), comprising mainly macrophages like Kupffer cells (KCs) and liver sinusoidal endothelial cells (LSECs) [9]. Both cell types are well recognized as protagonists in the modulation of liver inflammation [10,11]. For years we and others have evaluated strategies to load NPs with DXM aiming to decrease liver inflammation, while avoiding systemic adverse effects [12,13,14,15,16]. Nevertheless, no DXM-loaded NP has been successfully transferred to the clinic so far [17]. The most common difficulties associated with encapsulation of relatively small molecules like DXM are low encapsulation efficiency, poor formulation stability and unsuccessful drug release kinetics [18,19].

In this context, solid lipid nanoparticles (SLNs) have been described as stable and well-tolerated NPs for drug delivery [20]. The development of SLNs introduced advantages over other lipid-based nano-carriers, such as the avoidance of non-aqueous solvents during the synthesis process and the easy access to upscaling processes [21]. Despite these beneficial characteristics, low drug loading efficiency and poor stability over time limited the application of SLNs as drug carriers [22]. To overcome such limitations, a second generation of lipid nanoparticles, called nanostructured lipid carriers (NLCs) was developed [23,24]. Notably, during the synthesis process, the incorporation of a liquid lipid generates an amorphous zone inside the crystalline matrix of the lipid nanoparticle. In this regard, creating a less ordered solid lipid matrix leads to a higher drug loading efficiency. Drug molecules self-arrange in the spaces between fatty acid chains and in the amorphous areas of the liquid lipid, forming drug clusters [22]. We have recently demonstrated the flexibility and reliability of an NLC platform, by reporting on the suitability of multiple NLC-based formulations for the delivery of different types of drugs [25,26,27,28,29,30,31,32,33].

It is worth mentioning that DXM is generally considered to be a hydrophobic drug [34,35]. Thus, the hydrophobic nature of NLCs could potentially provide a notable environment for DXM delivery. In the study presented here, a novel NLC formulation has been developed for the encapsulation and sustained release of DXM (NLCs-DXM). By changing the liquid lipid ratio within the formulation, we obtained candidates with different DXM encapsulation efficiencies and physicochemical characteristics. The top candidate showed high DXM loading, persistent stability over time, and sustained DXM release, as well as negligible interaction with plasma proteins that could diminish the in vivo circulation time. We further observed no hemotoxic effects of these NLCs-DXM in human blood. Moreover, functional effects of NLCs-DXM were evaluated in primary cultures of murine NPCs. NLCs were internalized by NPCs in a dose-dependent manner. DXM released from NLCs showed pharmacological activity, as evidenced by decreasing levels of inflammatory cytokines (IL-6 and TNF-α) in LPS-stimulated NPCs. As another indication of anti-inflammatory effects, both soluble DXM and NLCs-DXM decreased levels of the conventional activation marker CD86 in stimulated LSECs and DCs. Finally, delayed release of biologically active DXM from NLCs was confirmed in time kinetics studies using a NF-κB reporter cell line. Taken together, these findings suggest that NLCs could provide a reliable platform for the delivery of DXM to the liver. This approach would be beneficial for improving the treatment of pathologies associated with chronic liver inflammation.

## 2. Materials and Methods

### 2.1. Materials

Myristyl myristate (Crodamol^TM^, MM) and the oil (Crodamol^TM^ GTCC-LQ) were donated by Croda (Buenos Aires, Argentina). Poloxamer 188 (Pluronic^®^ F68) and Dexamethasone (DXM) were purchased from Sigma-Aldrich (Buenos Aires, Argentina). All other reagents (buffers, solvents) were of analytical grade.

### 2.2. Preparation of DXM Loaded Nanostructured Lipid Carriers (NLC)

DXM-loaded NLCs were prepared by the hot-homogenization and ultra-sonication method, based on previous protocols from our laboratory [28,29]. Briefly, 400 mg of MM lipid (2.0% *w*/*v*, weight/volume; melting point 36–40 °C) was melted at 60–70 °C in a water bath, followed by the addition of 10 mg of DXM. Different amounts of liquid oil ranging from 0–100 µL were added to the lipidic phase to produce the different NLC formulations (F1–F4). After ten minutes, a pre-warmed aqueous solution (20 mL) containing 3.0% *w*/*v* poloxamer 188 was added to the melted lipid phase. Then, the mixture was sonicated in an ultrasonic processor at 80% amplitude for 30 min (130 W, Cole-Parmer, Vernon Hills, IL, USA; 6 mm titanium tip). The dispersion was cooled first at room temperature, and then stored at 4 °C.

### 2.3. Analytical Detection of DXM

The analytical determination of DXM in the range of 0–50 µg/mL was performed by UV-Vis spectrophotometry in a microplate reader (TECAN Infinite 200 PRO, Crailsheim, Germany) at λ_max_ = 242 nm. 

### 2.4. Determination of Encapsulation Efficiency (EE) and Drug Loading (DL)

After NLCs were prepared, 500 μL of the formulation was transferred to an ultrafiltration centrifugal device (MWCO 3000, Microcon, Millipore, MA, USA) and centrifuged at 10,000× *g* and 25 °C for 10 min in a microcentrifuge (Eppendorf MiniSpin, Hamburg, Germany). The DXM concentration in the filtered solution was determined by UV-Vis spectrophotometry (microplate reader TECAN Infinite 200 PRO, Männedorf, Switzerland). The EE and DL were calculated as follows:

(1) $$\mathrm{EE}\;(\%)=\frac{(Q_{0}-(C_{r}\times V))\times 100}{Q_{0}}$$

(2) $$\mathrm{DL}=\frac{\mathrm{Mass}\;\mathrm{of}\;\mathrm{encapsulated}\;\mathrm{DXM}\;\left(\mu g\right)}{\mathrm{Mass}\;\mathrm{of}\;\mathrm{MM}\;\mathrm{lipid}\;\mathrm{matrix}\;\left(\mathrm{mg}\right)}$$

Q_0_ (initial mass of DXM); C_r_ (DXM concentration measured in the filtered solution); V (final volume of the formulation).

### 2.5. Particle Size, Zeta Potential (Z pot), and Polydispersity Index (PDI)

The mean diameter, size distribution (PDI), and zeta potential (Z pot) of the NLC formulations were determined in a Nano ZS Zetasizer (Malvern Instruments Corp., Malvern, UK) in polystyrene cuvettes with a path length of 10 mm at 25 °C and in capillary cells with a path length of 10 mm, using Milli-Q water. All measurements were carried out in triplicate. 

The stability of the formulations was followed up by testing particle size, Z pot, and NLC drug content (EE) for up to 12 months. 

### 2.6. Transmission Electron Microscopy (TEM)

The formulations were diluted with Milli-Q water (1:10), and a drop of the dispersion was spread onto a 400-mesh collodion-coated Cu grid (Merck KGaA, Darmstadt, Germany). Phospho-tungstic acid was added to enhance contrast. Images were observed in a Jeol-1200 EX II-TEM microscope (Jeol, MA, USA).

### 2.7. In Vitro Release of DXM

The various NLC formulations were placed into dialysis devices (1 mL; Spectra-Por^®^ Float-A-Lyzer^®^ G2, MWCO ¼ 100 kDa), followed by incubation in a Falcon tube with 30 mL of 10 mM phosphate buffer saline (PBS) at pH 7.4 and 37 °C. Additionally, a DXM solution with equivalent concentration as formulations was prepared to estimate the diffusive effect through the dialysis device. The release assays were performed in an orbital shaker (DLAB-HM100 Pro) at 100 rpm. Samples of 300 µL were retrieved and scanned with an UV-Vis spectrophotometer (TECAN-infinite M200 Pro) at intervals of 1, 2, 3, 4, 5, 6, and 24 h to determine concentrations of released DXM. The fitting curve tool of SigmaPlot 10.0 Notebook program (Systat Software Inc., San José, CA, USA) was used to fit the experimental data according to four release-mechanism models from the fit library.

### 2.8. Hemotoxicity Studies

Heparinized venous blood was obtained from healthy donors after written informed consent. It was centrifuged at 2500× *g* for 10 min. The plasma was discarded, and the erythrocyte concentrate was washed 3 times with PBS. Erythrocytes were diluted 1:4 in PBS. A volume of 2.0 mL was incubated with different concentrations of NLC formulations at 37 °C for 1, 24, and 72 h. After incubation, the mixture was centrifuged at 2500× *g* for 5 min and the precipitate was discarded. The percentage of lysed erythrocytes was determined by quantification of released hemoglobin at λ = 540 nm. The 100% hemolysis value was established by treatment of erythrocytes with 1%Triton X-100 for 1 h, and the negative control by incubation of erythrocytes with PBS.

### 2.9. In Vitro Interaction with Plasma Proteins

In order to investigate the biomolecular interactions that may occur between NLCs- DXM and plasma proteins, surface plasmon resonance (SPR) measurements were performed. In the present work, immunoglobin (IgG) and fibrinogen were tested as examples of opsonins (shown to diminish circulation time) [36] and human serum albumin (HSA) as a disopsonin (reported to enhance circulation time) [37].

Protein was immobilized by physical adsorption [38] by injecting protein solutions into a SPR Navi^®^ 210A (BioNavis, Tampere, Finland) instrument operated at 22 °C and with λ= 785 nm. Samples of HSA, IgG and fibrinogen (each 100 µg/mL) in 10 mM sodium acetate (pH 4.4) were injected (10 µL/min, 20 min) through flow cell 1 over the sensor chip in each three different individual experiments. Control surfaces were obtained by injection of buffer solution w/o protein (flow cell 2). The SPR response (Δϴ_SPR_) was measured as the difference between the ϴ_SPR_ value after protein injection and the ϴ_SPR_ value of the baseline (before injection).

Interactions of immobilized protein with NLCs were studied by injecting a ten-fold dilution of NLCs-DXM in PBS (pH 7.4) using the SPR setup (10 µL/min, 10 min) as described above, followed by a regeneration step using 1% Triton X100 (100 µL/min, 1 min). The SPR response assigned to biomolecular interaction corresponded to the plateau of Δϴ_SPR_ in the period between the end of the NLCs-DXM injection and the start of the regeneration step (around 720 s of each cycle).

### 2.10. Isolation of Liver Non-Parenchymal Cells (NPCs) 

NPCs were isolated from livers of female C57BL/6J mice as previously reported [16,39]. Collected NPCs were centrifuged at 300× *g* and resuspended in ice-cold Histodenz solution (30%). Another centrifugation step at 1500× *g* at 4 °C for 20 min was performed. NPCs were collected at the HBSS/Histodenz interface using a Pasteur pipette and resuspended in RPMI 1640 medium containing 10% FCS, 1% penicillin/streptomycin, 1 mM L-glutamine, 1% essential and non-essential amino acids, 50 μM 2-mercaptoethanol (all media, buffers, and reagents from ThermoFisher Scientific, Waltham, MA, USA). 

### 2.11. Flow Cytometry 

After cell isolation, NPCs were incubated with receptor-specific antibodies for flow cytometry analysis. Cells were washed (2% FCS in PBS), and Fc receptors were blocked with a CD16/CD32-specific antibody (clone 2.4G2) for 10 min. Afterwards, NPCs were incubated with cell lineage-specific antibodies (CD45-BV711, CD32b-PE, CD11c-PE-Cy7, F4/80-eFluor450) for 20 min at 4 °C. Next, cells were washed (2% FCS in PBS) and live/dead markers (FVD-780, 7AAD) were added. Cells were washed again and subjected to flow cytometry (Attune NxT Flow Cytometer, Thermo Scientific, Waltham, MA, USA). Results were analyzed with Attune NxT Flow Cytometer Software.

### 2.12. Cytometric Bead Array

NPC culture supernatants were collected and stored at −20 °C. To measure TNF-ɑ, IFN-γ, IL-6, IL-1ß, and IL-10, Cytometric Bead Assays were performed, as recommended by the manufacturer (BD Biosciences, CA, USA). Recombinant cytokines were used to prepare standard dilutions. Samples were mixed with capture beads and subsequently incubated with PE-conjugated detection antibodies for 1h (all at room temperature in the dark) and measured by flow cytometry. Results were analyzed using FCAP Array Analysis Software v.1.0.1 (BD Biosciences).

### 2.13. Detection of Nuclear Factor ‘Kappa-Light-Chain-Enhancer’ of Activated B-Cells (NF-κB) in a Raw-Blue™ Reporter Cell Line

The macrophage reporter subline RAW-Blue™ (InvivoGen, San Diego, CA, USA) was used to determine NF-κB activity. RAW-Blue™ cells express secreted embryonic alkaline phosphatase (SEAP) which is inducible by active NF-κB. Raw-Blue^TM^ cells were incubated with 1000 µg/mL of DXM and equimolar amounts of NLC-encapsulated DXM as indicated and stimulated with LPS applied 45 min later. Supernatants (20 µL) were collected 6h and 24h after LPS stimulation. All samples were treated in parallel with Quanti Blue solution (180 µL; InvivoGen) serving as SEAP substrate according to the vendor. Finally, NF-kB activity was determined as substrate turnover by colorimetric analysis in a spectrophotometer (EMax^®^ Pus Microplate Reader; Molecular Devises, San José, CA, USA) at 620–655 nm.

### 2.14. Statistical Analysis

Statistical analyses were performed with Prism software (Graphpad Software, San Diego, CA, USA) performing one- and two-way ANOVA as indicated. Data are presented as the means ± SEM. *p*-values < 0.05 were considered significant.

## 3. Results

### 3.1. Preparation and Characterization of DXM-Loaded NLCs

NLCs matrices combining different amounts of solid lipid with liquid oil were prepared by the ultra-sonication method [25]. The solid lipid matrix was mainly composed of MM. MM was selected as the main building block of the NLC structure, due to its low melting point (of around 40 °C), which is suitable to preserve the integrity of the encapsulated drugs and to prepare stable emulsions. MM acts as a co-emulsifier by thickening the emulsions, due to the combination of fatty alcohol and fatty acids that prevent instability caused by temperature variations. Moreover, MM is a natural product extracted from plants and has been reported to be non-toxic [25]. The addition of an inert oil (GTCC-LQ) to the NLCs’ matrices aimed to modify the crystalline structure of the SLNs, without affecting their biological properties. This hypothesis was supported by previous reports from our group demonstrating that combinations of MM/GTCC-LQ oil resulted in the formation of non-toxic NLCs [28]. In the current study, stable NLC formulations were obtained by incorporating different amounts of the liquid oil into the synthesis mixture. NLCs were further characterized by the determination of EE and other physicochemical parameters, as described in Table 1. The F1 formulation, formally SLNs because of the absence of oil, showed an EE of 86% with a mean size around 152 nm, low PDI, and negative surface charge. The addition of 25 µL of oil to the lipid phase, generated NLCs (F2) with an increased EE of 98%, a mean diameter of 145 nm, and a Z pot 5.0 mV less negative than the measured for F1. In the cases of F3 and F4, increasing the amount of oil added to the MM lipid did not significantly affect the physicochemical properties of these NLCs in comparison with F2. In all cases, drug loading ranged from 21 to 25 µg of DXM per mg of MM lipid. The PDI values of all prepared formulations were below 0.3. 

**Figure 1:**
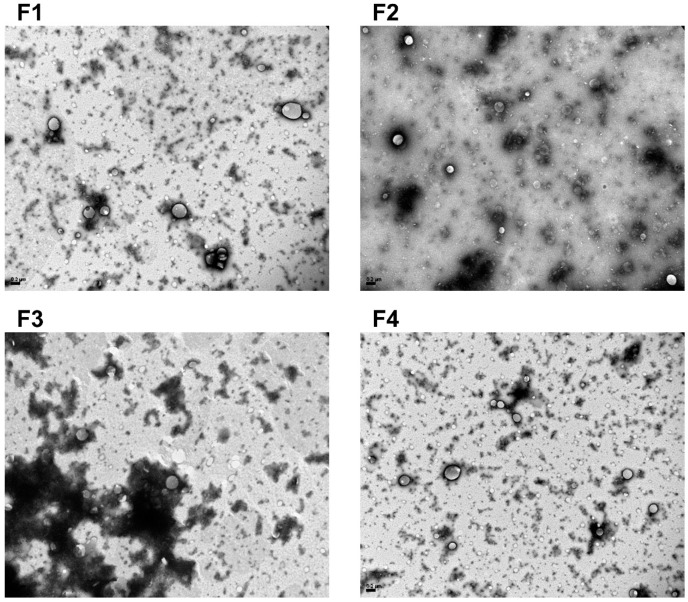
TEM images of NLCs (F1 to F4 formulations). The freshly prepared formulations (20 mg MM lipid/mL) were diluted with Milli-Q water (1:10) and the contrast was enhanced with phospho-tungstic acid. All images were taken at 30,000× (scale bar = 200 nm).

The morphology of the different formulations was determined by TEM (Figure 1). Clear spherical NPs presented with a narrow PDI. In correlation with the results of previous studies presented above, the addition of liquid oil to the lipid matrix did not affect NLC morphology. In all images, the presence of small micelles of Poloxamer 188 could be visualized. No aggregates or precipitates of NLCs were observed, suggesting stability of the formulations. 

### 3.2. DXM Release from NLCs

**Figure 2:**
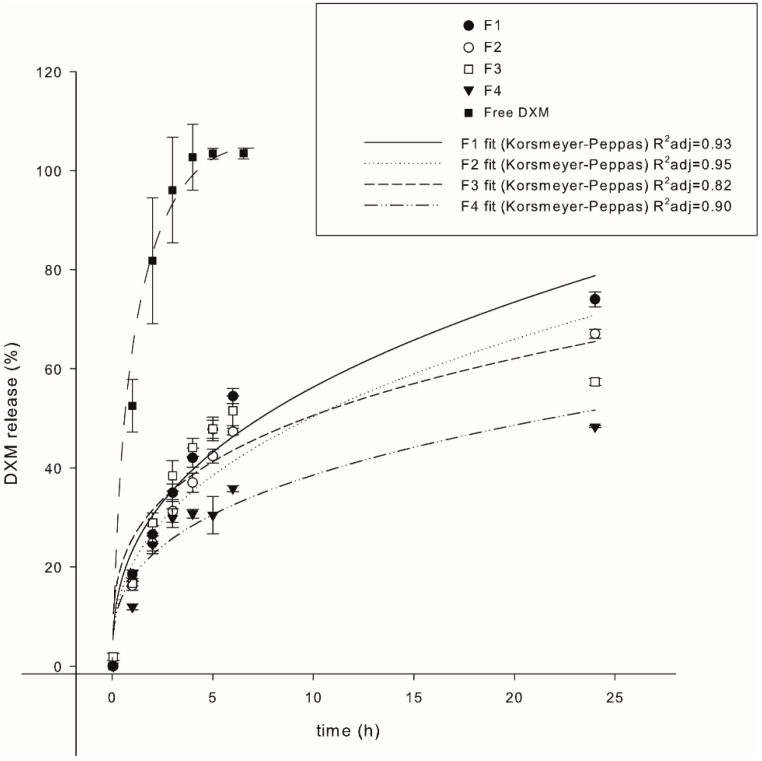
DXM release inversely correlates with oil content of the NLC matrix. Time-dependent DXM release from the different NLC formulations in 10 mM PBS pH 7.4 and 37 °C, (*n* = 3).

DXM release by NLC formulations was determined to study drug kinetic mechanisms (Figure 2). All formulations showed a typical bi-phasic release profile, with a burst release during the first 3 h followed by a sustained release at a lower rate. Additionally, all formulations showed a longer release profile compared to the soluble DXM control. An increase in the content of liquid oil in the matrix resulted in a slower release of DXM. Thus, 74.0% (F1), 67.1% (F2), 57.4% (F3), 48.3% (F4) of DXM was released in the course of a 24 h incubation, respectively. In particular, F4 released less than 40% of the drug within 12 h. Formulation-specific DXM release kinetics were fitted with different established models to delineate drug release mechanisms: First Order, Higuchi, Korsmeyer-Peppas, and Hixon and Crowell (Table S1). The release model that fitted best to the experimental data was selected according to the calculations of the coefficient of determination (R^2^). The release of DMX from all tested formulations matched with the Korsmeyer-Peppas model (Figures S1–S4) [40]. The *n*-value is indicative of the mechanisms involved in drug release from the matrix. The adjustments resulted in the determination of release exponents (“n”) lower than 0.5 for the various NLC formulations. The experimental data values (dots)according to Korsmeyer-Peppas fitting curves (lines) are depicted in Figure 2.

### 3.3. Stability after Storage

**Figure 3:**
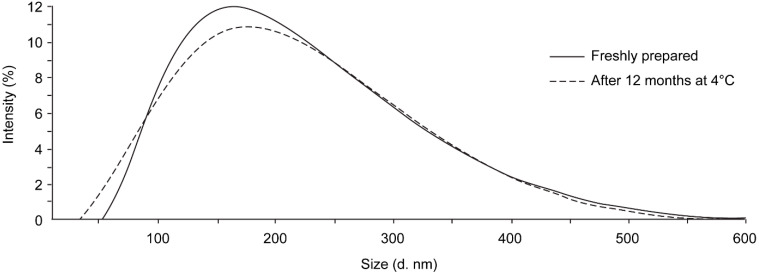

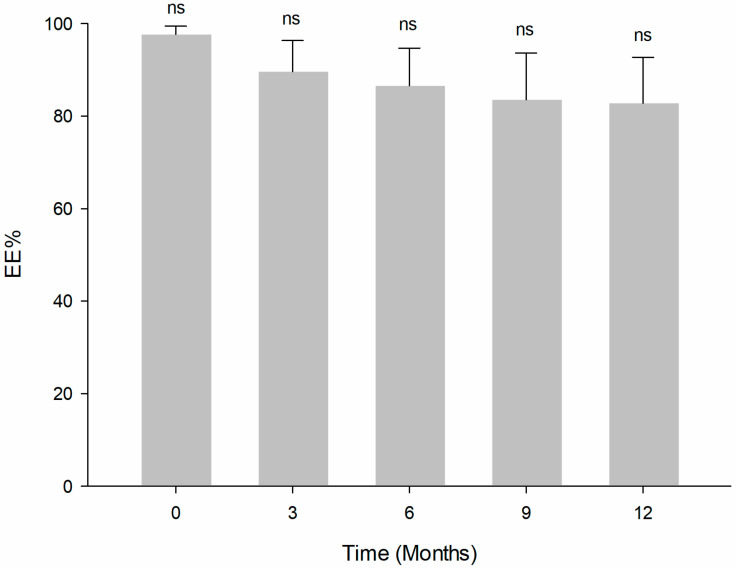
**NLC formulations are stable over time.** Stability of NLCs-DXM formulation after 12 months stored at 4 °C. **Upper panel**: size distribution- Original sample: Mean diameter: 145.0 ± 0.8 nm; PDI: 0.20 ± 0.01; older formulation: size: 150.8 ± 2.5 nm; PDI: 0.24 ± 0.02; **lower panel**: evolution of the EE measured every 3 months, (*n* = 3); no significant difference (ns) (Two-way ANOVA, Dunnett’s multiple comparisons test).

Based on the results of the comprehensive analyses of the different NLC formulations, F4 was selected as the top candidate for further evaluations (from now on called NLCs-DXM). This NLC-DXM formulation was stable for at least 1 year when stored at 4 °C. No significant changes in size distribution and PDI were observed (Figure 3, upper panel). The Z pot values of freshly prepared NLCs and 1 year-stored NLCs were −9.3 ± 1.4 mV and −12.0 ± 0.4 mV, respectively, suggesting no significant changes (*p* ≥ 0.05). Furthermore, no significant differences were noted regarding the NLC drug content (EE) measured every 3 months for a total of 1 year (Figure 3, lower panel).

### 3.4. Hemotoxicity Studies

**Figure 4:**
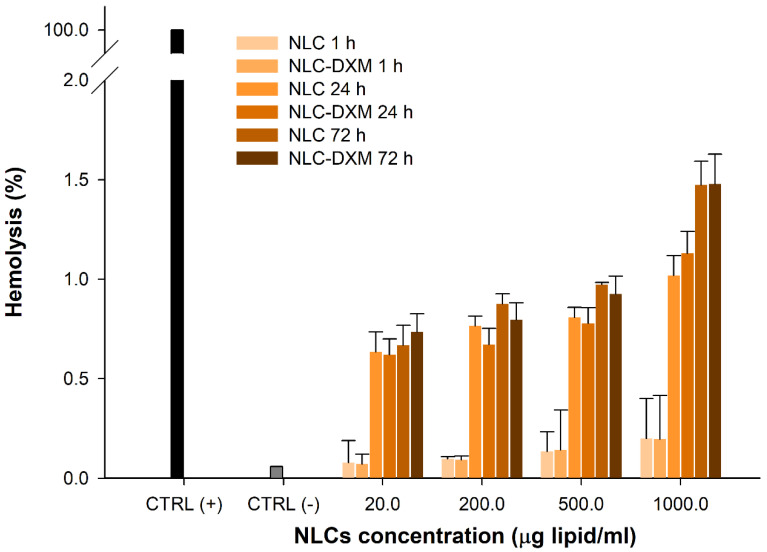
**At high concentrations NLCs induce only minimal hemotoxicity.** Hemotoxicity of empty and DXM loaded NLCs at different concentrations after 1, 24 and 72 h incubation. Controls: (−) untreated; (+) 1% Triton X-100-lysed erythrocytes, (*n* = 3).

Next, the hemocompatibility of NLC formulations was assayed (Figure 4). Different concentrations in the range from 20.0 to 1000.0 µg MM lipid/mL were tested. Negligible hemotoxic effects (<0.4%) were observed when applying both NLCs and NLCs-DXM formulations (1 h) to erythrocytes at the highest NLC concentration tested (1.0 mg/mL). After 24 h incubation, a low degree of hemolysis was observed with values below 1.1%. After 72 h incubation at the highest NLC concentration, about 1.5% of erythrocytes were lysed by either NLC formulation.

### 3.5. Interactions of DXM-Loaded NLCs with Serum Proteins

The interaction of NLCs-DXM with three different plasma proteins commonly found in the protein corona of NPs was studied by SPR experiments. In this regard, potential interactions with HSA, IgG and fibrinogen were tested. Net changes in ϴ_SPR_ were observed after the injection of the protein solutions onto the SPR chip, verifying the physical adsorption of HSA, IgG and fibrinogen (ΔΘ_SPR_ of 0.25, 0.23 and 0.43 degrees, respectively). 

**Figure 5:**
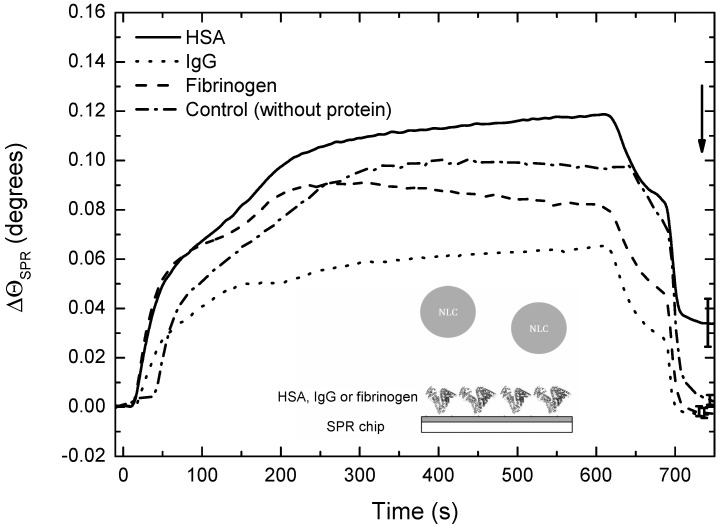
**HSA, but no opsonin interacts with NLCs.** SPR studies to determine interactions of DXM-NLCs with serum protein having HSA, IgG and fibrinogen. All sensor-grams are presented as mean ± SEM of 3 independent experiments. The arrow indicates the final time of the experiment, when interactions were analyzed.

SPR sensor-grams obtained after the injection of NLCs-DXM onto protein-modified chips or control surfaces are shown in Figure 5. They verified a minimal Δθ_SPR_ (0–0.005) in the case of bare gold or opsonin-covered sensor surface [24]. NLC-DXM, immobilized had Δθ_SPR_ (~0.035), and evident in vitro interaction was revealed.

### 3.6. NLCs Binding to Liver NPC Populations 

**Figure 6:**
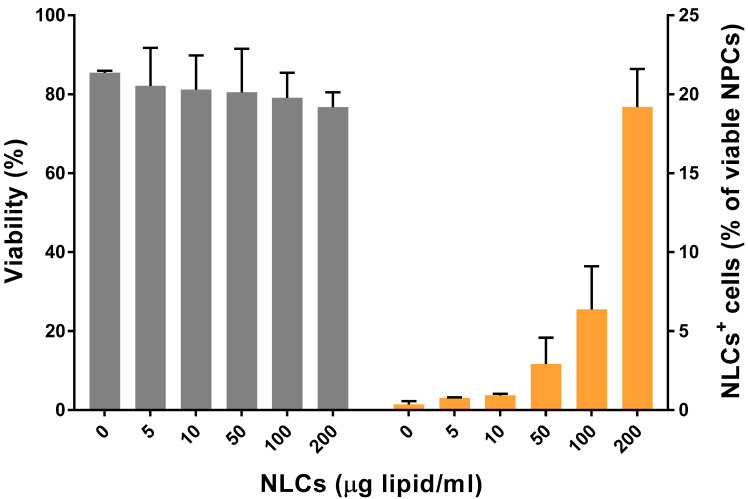
**NLCs are internalized by NPC in a dose-dependent manner and are devoid of cytotoxic activity.** NPCs were incubated for 24 h with NLCs at concentrations between 0 and 200 µg/mL to evaluate potential cytotoxic effects (left *y*-axis). In the same concentration range, DiD-labeled NLCs were used to study cellular uptake (right *y*-axis). NLCs concentrations are expressed as µg of MM (solid lipid) per mL. Data are the means ± SEM obtained in 3 independent experiments.

The biological activity of NLC formulations was tested using primary murine NPCs as potential target cells for DXM delivery. NPCs were incubated for 24 h with NLCs at different concentrations ranging from 0 to 200 µg of solid MM lipid/mL of culture media. Viability assessment showed no apparent cytotoxic effects of NLCs on NPCs after incubation (Figure 6). Furthermore, NLC uptake was evaluated using NLCs labeled with DiD dye. Flow cytometry results demonstrated a dose-dependent uptake of NLCs by NPCs, up to 20% in case of the highest dose applied.

### 3.7. DXM Functionality Is Not Affected by Encapsulation into NLCs

**Figure 7:**
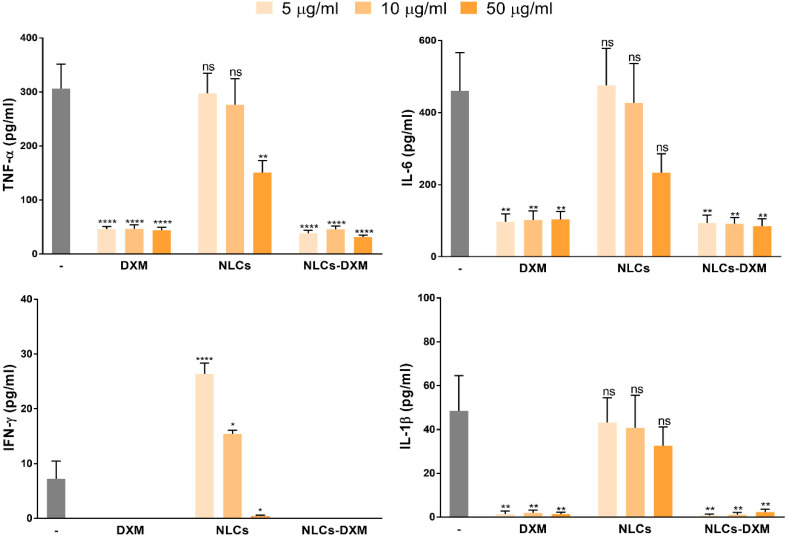
**NLC-derived DXM prevents LPS-induced NPC activation.** TNF-α, IL-6, IFN-γ and IL-1β levels of DXM-treated of NPCs cultures were monitored. NPCs were incubated with NLC formulations (5, 10 and 50 µg/mL), followed by LPS (100 ng/mL) application. On the next day, supernatants were subjected to cytokine analysis. Data denote the means ± SEM obtained in 3 independent experiments. Significantly different from control (LPS only): no significant difference (ns); * *p* < 0.05; ** *p* < 0.01; **** *p* < 0.0001 (Two-way ANOVA, Dunnett’s multiple comparisons test).

Due to the high DXM loading capacity of NLCs, only the concentration range between 5 and 50 µg of solid MM lipid/mL of culture media was tested further. Freshly isolated NPCs were incubated with equimolar amounts of DXM in its soluble and particulate (NLCs-DXM) forms, and empty NLCs (same dose as for NLCs-DXM). LPS was applied 45 min later to trigger pro-inflammatory responses. After 24 h incubation, supernatants were retrieved for cytokine measurements. The results showed that soluble DXM at all concentrations tested reduced pro-inflammatory cytokine levels, as compared to the positive control (LPS only) (Figure 7). Notably, NLCs-DXM induced a similar response as soluble DXM. Interestingly, empty NLCs also affected cytokine secretion in comparison with the control in several cases. Whereas IL-1β levels were not altered upon preincubation of liver NPC with NLCs prior to LPS stimulation, a significant decrease in TNF-α production was observed at the highest NLC concentration. A similar tendency was noted in the case of IL-6. In contrast, NLCs at low and intermediate concentrations further increased LPS-induced IFN-γ production.

### 3.8. NLCs Induce Anti-Inflammatory Effects Evidenced by the Reduced Activation State of NPCs Subpopulations

**Figure 8:**
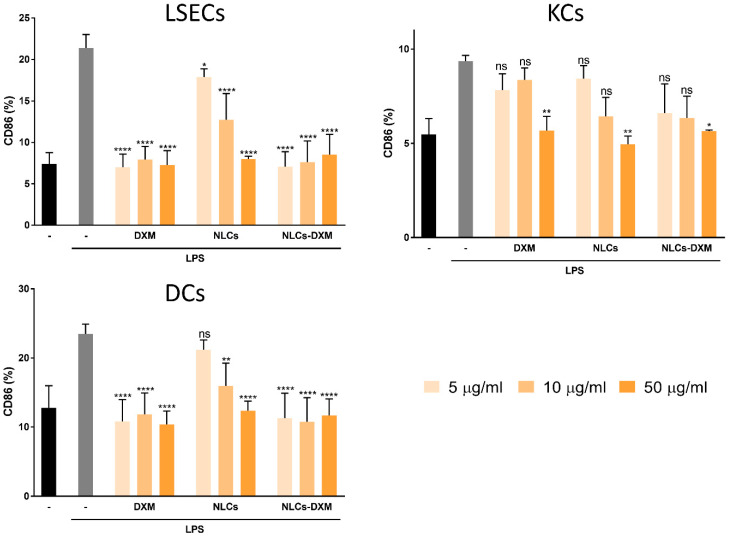
**DXM released from NLCs counteracts induction of CD86 by LPS.** NPC cultures were treated with soluble DXM, empty NLCs and DXM-loaded NLCs at three different concentrations (5, 10 and 50 µg/mL). LPS (100 ng/mL) was applied 45 min later. On the next day, NPC populations were phenotypically characterized: LSECs (CD45^+^ CD32b^+^), KCs (CD45^+^ F4/80^+^) and DCs (CD45^+^ CD11c^+^). The activation state of each cell type was studied by CD86 marker assessment. The gating strategy has been described [39]. Data represents the mean ± SEM obtained in 3 independent experiments. Significantly different from positive control (LPS only): no significant difference (ns); * *p* < 0.05; ** *p* < 0.01; **** *p* < 0.0001 (one-way ANOVA, Dunnett’s multiple comparisons test).

The cellular activation state was further evaluated for each of the NPC subpopulations. As expected, LPS treatment induced an up-regulation of the co-stimulatory marker CD86 by either NPC population. However, when NPCs were pre-treated with soluble DXM the frequency of CD86^+^ cells reduced. This decrease in cellular activation confirmed the anti-inflammatory effects of DXM for each of the NPC populations studied. Moreover, very similar effects were observed in the case of treatment with NLCs-DXM (Figure 8). Empty NLCs also prevented the LPS-induced upregulation of CD86 by either liver NPC population, but only in the case of the highest dose. However, in KCs only the highest concentration of DXM in soluble or particulate form significantly attenuated LPS-induced CD86 upregulation. In contrast, LSECs and DCs displayed total inhibition of LPS-induced CD86 upregulation when treated with DXM in soluble or particulate form, in either case even at the lowest dose (5 µg/mL) applied. 

### 3.9. Delayed Release of DXM from NLCs Affects LPS-Induced NF-kB Activity in a Time-Dependent Manner

**Figure 9:**
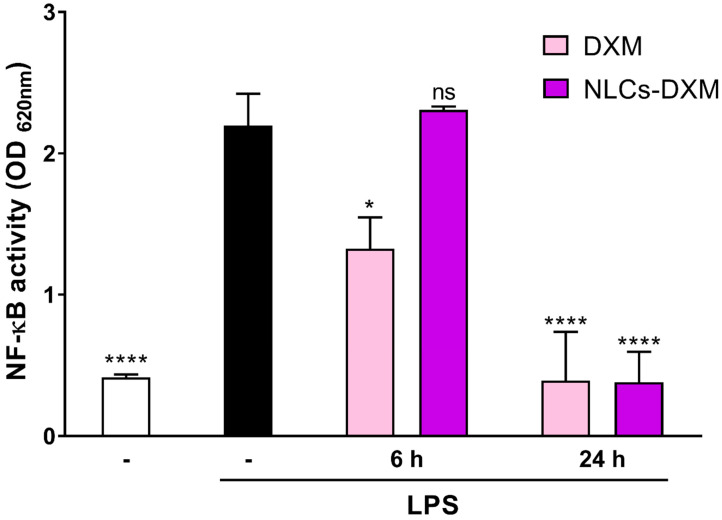
**NLCs-DXM inhibit LPS stimulation due to delayed release of the drug.** RAW-Blue™ cells, derived from RAW 264.7 macrophages and containing SEAP reporter construct inducible by NF-κβ, were pre-treated with DXM or NLCs-DXM (each 1000 µg/mL). Next, LPS was added to the culture media to induce NF-κβ activation. DXM-mediated inhibition of this response by both treatment options was measured by NF-κβ-dependent, SEAP activation. Data represent the means ± SEM obtained in 3 independent experiments. Significant differences versus LPS^+^ untreated cells are indicated: no significant difference (ns); * *p* < 0.05; **** *p* < 0.0001 (Two-way ANOVA, Dunnett’s multiple comparisons test).

To elucidate whether DXM was released in a sustained manner from NLCs a reporter cell line carrying a reporter construct with a NF-κB-inducible promotor was pre-incubated for 45 min with DXM or NLCs-DXM prior to LPS treatment and NF-kB activity was monitored after the indicated period of time. DXM treatment partially inhibited LPS induction of NF-κB when assessed 6 h later (Figure 9). In the case of NLCs-DXM treatment NF-κB activity by LPS was fully inhibited only at 24 h after the onset of incubation, similar to DXM-treated cells. This finding confirmed a slow release of DXM from NLC. 

## 4. Discussion

The incorporation of liquid oils into solid lipid matrices is an important step toward the formation of NLCs [21]. Previous work from our group has provided evidence that the combination of MM/GTCC-LQ oil resulted in the formation of non-toxic NLCs. Based on this previous study, we aimed to use NLCs for DXM delivery. Formulation F1 represented conventional SLNs without the addition of oil, which is necessary to produce the nanostructure of the matrix [22]. Incorporation of 1/16 ratio (vol./lipid weight) of oil into the lipid generated NLCs (F2) with a somewhat smaller diameter and less negative zeta potential than F1, suggesting a modification of the structure of the lipidic matrix due to the presence of the liquid oil [28]. The same tendencies were observed for formulations with higher oil content (F3, F4), but without significant differences in comparison to F2 (*p* > 0.05). The PDI of the formulations was very low, which suits biological applications [41]. Additionally, TEM results showed a narrow size distribution of the spherical NLCs in accordance with previous work from our laboratory that reported the stability of such colloidal systems [25]. 

Drug release kinetics from delivery devices constitute a critical point regarding the necessity to obtain desirable concentrations of that drug in a therapeutic range over a longer period of time [40]. In the case of DXM, the major challenge was to improve patient compliance by decreasing the dosing frequency to reduce side effects, but without losing therapeutic effectiveness. All comparatively assessed NLC formulations showed a typical bi-phasic release profile, with a burst release in the first hours followed by a sustained release afterwards. Different reports have described the same behavior for drugs encapsulated into SLNs/NLCs [31,42]. The fitting of experimental data with the different release model mechanisms demonstrated that the formulations F1 to F4 adjusted best to Korsmeyer-Peppas [40]. This semi-empirical model was originally developed to describe the release of drugs from polymeric matrices; however, it has also been used for other types of matrices as well [43]. In particular, the exponential parameter of the model “n” is useful to study the mechanism which determines the release behavior. For “n” < 0.5 the mechanism is considered to be diffusion following Fick’s law, while for higher “n” other mechanisms, such as matrix swelling, were found to be relevant [44]. For all formulations tested in this work, the release exponent “n” was found to be <0.5. This finding is consistent with the fact that NLC lipid matrices do not undergo swelling like polymeric matrices [45]. Several factors, like drug-lipid specific interactions, drug location inside nanoparticle and nanoparticle structure, were reported to govern the release of drugs from NLC systems. Mainly because of this, either extension or reduction in release intervals have been reported when modifying NLC structure with liquid oils [46]. As stated before, the inclusion of an oil in the lipid matrix prevents crystallization and the consequent expulsion of the drug from the nanoparticle. However, other mechanisms could explain the profiles observed in the release kinetics from the different formulations. The increase in the proportion of liquid oil could be generating “pockets” inside the NLC, moving the DXM away from the interface and delaying its release [46]. In this context, the slow and controlled release of DXM from formulation F4 was a very interesting result, since it may allow controlling DXM release for a prolonged period of time. This constitutes an important progress in controlling the release of DXM from NPs in comparison to previous studies. For instance, Xiang et al. showed that the extent of DXM released from SLNs was around 75% after 12 h [47]. In the work from Wang et al. a 40% release was obtained after 12 h [48], while Xu et al. reported a 70% release within 12 h [49], both using NLCs matrices. Altogether, formulations developed for DXM delivery have showed undesirable release profiles so far, since a considerable amount of the cargo was released rapidly after administration. The main characteristic of an according formulation should be a steady low-level release to prevent concentration-associated side effects.

Based on size distribution, EE and DXM release kinetics, formulation F4 was selected for further experiments. Remarkably, good stability of F4-derived NLCs-DXM was observed even after 12-month storage, which constitutes a great advance considering the need to ensure a reliable half-life of nanodrugs. Moreover, particle size distribution and zeta potential remained unaltered, which suggested no changes in crystallinity of the lipids during storage, conceivably attributed to the presence of the liquid oil into the matrix [50]. The low Z potential values observed in the formulation were due to stabilization with a non-ionic surfactant (Poloxamer 188). The steric groups of the surfactant stabilized the formulation by avoiding the agglomeration of nanoparticles in suspension [51,52]. Kovacevik et al., reported that the inclusion of a non-ionic surfactant in the formulation of NPs contributes to the stability of the formulation, even in NPs with ZP that does not reach the critical −30 mV [53]. Shah et al., referred to the physical stability of nanoparticles depending on many other factors that contribute to producing stable formulations. In this regard, NLCs in suspension with a non-ionic surfactant showed stability despite the lower zeta potential (and less electrostatic stabilization), but with great steric stabilization [54].

Nanodrugs frequently interact with serum factors which may selectively adsorb to the surface of the nano-carrier and, thereby, control its cellular interaction [55]. Therefore, it is necessary to study potential interactions that NLCs may establish with biomolecules and cells [56]. In the case of intravenous administration, NLCs-DXM may contact erythrocytes. In fact, some types of NPs have been reported to exert inflammatory responses and hemolysis [57]. According to ISO/TR 7406, biomaterials that induce a critical hemolytic ratio < 5% are considered safe for human applications [58]. Our results demonstrated that NLCs-DXM did not cause hemolysis exceeding this level, even after 72 h exposition, suggesting the absence of potential hemotoxic side effects. Another parameter to study was the protein corona of the nano-carrier, which can mask its chemical identity and affect its biodistribution [59]. SPR experiments verified that the in vitro biomolecular interaction between NLCs-DXM and the opsonins IgG or fibrinogen was negligible, whereas NLCs-DXM readily engaged HSA, which may enhance the in vivo circulation time of the NP.

Most likely, after entering systemic circulation, NLCs-DXM will end up in the liver, which constitutes an intended target organ for the delivery of DXM. Among the wide range of DXM applications, the treatment of liver inflammation with this anti-inflammatory agent has been reported in detail [60,61]. Therefore, we studied the functional effects of NLC formulations using liver NPCs. This heterogeneous cell population confers most immune-related responses within the liver [9]. NPCs have the capacity to react towards exogenous stimuli, such as antigenic compounds or danger signals like LPS. The latter, mediated by TLR4 binding, induces proinflammatory responses [62]. In this study, NPCs were pre-treated with DXM to induce an anti-inflammatory state, followed by LPS application. Notably, NPC viability was not affected after 24 h incubation with NLCs. Besides this, NLCs uptake by NPCs proved to be successful within the concentration range tested. This might be mainly associated with the particular endocytic phenotype of the different NPC cell types [9]. Furthermore, the reduction of proinflammatory cytokine levels in the case of DXM and NLCs-DXM treatment proved that DXM released from the NLCs preserved its pharmacological activity. Remarkably, empty NLCs at the concentration of 50 µg/mL also partially inhibited cytokine secretion. These findings prompted us to perform more detailed analyses of the effects generated by NLCs on the NPC subpopulations. Thus, LSECs, KCs, and DCs were separately characterized phenotypically. As expected, NPCs reacted to LPS treatment by upregulating CD86 expression in all analyzed NPC types. Most importantly, NLCs-DXM applied before LPS prevented an increase in the frequencies of CD86^+^ cells, similarly to soluble DXM. Surprisingly, the highest tested concentration of empty NLCs also inhibited CD86 upregulation to a similar extent, as observed for DXM treatment. These results confirmed that NLCs could potentially have some intrinsic inhibitory effects on liver NPC population. In a similar way, other lipid-based nanocarriers were reported to have intrinsic anti-inflammatory effects, due to macrophage engagement generating an inhibition on proinflammatory cytokine secretion, both in vitro and in vivo [63,64]. Furthermore, NPs of other natures, such as gold NPs, quantum dots, silica NPs and food-derived NPs, were found to affect DC maturation showing inhibition of activation marker upregulation after LPS challenge [65,66,67,68]. Further experiments will be necessary to study, in more detail, the potential effects associated with NLC engagement by NPCs.

Finally, the question of whether NLCs-DXM released DXM in a sustained way also under cell culture conditions was addressed by using the RAW-Blue™ NF-κβ reporter cell line. Our results showed that, contrary to the inhibition of LPS-induced NF-κβ activity by soluble DXM assessed 6h after DXM/LPS application, treatment with NLCs-DXM had no effect after short time incubation. At the 24 h time point, however, NLCs-DXM inhibited NF-kB activity to a comparable extent as directly applied DXM, which might be a consequence of almost complete DXM release.

In summary, the following conclusions can be drawn: (1) NLCs composed of MM and GTCC-LQ oil show superior EE, stability and DXM release kinetics; (2) NLCs are well tolerated by human erythrocytes; (3) NLCs show negligible in vitro interaction with plasma opsonins; (4) Liver NPCs are capable of internalizing NLCs in a dose-dependent manner; (5) DXM released from NLCs preserves its anti-inflammatory activity.

Taken together, these findings provide evidence that the NLC platform introduced in this study has the potential to serve as a well-tolerated nano-carrier for the delivery and sustained release of bioactive DXM to the liver, potentially reducing systemic side effects otherwise associated with systemic DXM application in the treatment of liver inflammation.

## Figures and Tables

**Table 1:** Characteristics of NLC formulations.

*Sample*	*Composition*			*EE ^a^* (%)	*DL ^b^*	*Mean Diameter* (nm)	*PDI*	*Z Pot ^c^* (mV)
	MM (mg)	DXM (mg)	Oil (µL)					
*F1*	400	10	0	86.4 ± 3.0	21.6	152.2 ± 2.8	0.24 ± 0.01	−15.9 ± 1.0
*F2*	400	10	25	97.7 ± 3.1 *	24.4	144.9 + 1.0 *	0.22 ± 0.01	−10.4 ± 0.8
*F3*	400	10	50	94.3 ± 2.9 *	23.6	142.1 + 1.9 *	0.20 ± 0.01	−9.1 ± 0.4
*F4*	400	10	100	94.2 ± 3.0 *	23.6	145.0 + 0.8 *	0.20 ± 0.01	−9.3 ± 1.4

The results express the mean ± SD (*n* = 3). ^a^ Encapsulation efficiency. ^b^ Drug loading expressed as µg of DXM/mg of MM lipid. ^c^ Zeta potential, * *p* < 0.05 in comparison with F1 (SLNs-DXM).

## Data Availability

The data that support the findings of this study are available from the corresponding authors, M.L.C. and G.A.I., upon request.

## Supplementary Materials

The following supporting information can be downloaded at: https://www.mdpi.com/article/10.3390/pharmaceutics14081611/s1, Table S1: Calculations of the coefficient of determination (R^2^) for DXM release. Figures S1–S4: DXM release fitting curves with the Korsmeyer-Peppas model from all tested formulations.
