# Supplementary material for: Nanostructured Lipid Carriers Loaded with Dexamethasone Prevent Inflammatory Responses in Primary Non-Parenchymal Liver Cells
Source: Pharmaceutics. 2022 Aug 2;14(8):1611. doi: 10.3390/pharmaceutics14081611 (PMC9413549; doi:10.3390/pharmaceutics14081611)
Supplement: Supplementary file 1 [file pharmaceutics-14-01611-s001.zip › pharmaceutics-1821756-supplementary.pdf]

Article

Nanostructured Lipid Carriers Loaded with Dexamethasone Prevent Inflammatory Responses in Primary Non-Parenchymal Liver Cells

Carolina Medina-Montano & Ignacio Rivero Berti et al.

Supplementary Material

Table S1. DXM release kinetics of the different formulations were fitted with different models used for the description of drug release mechanisms (First Order, Higuchi, Korsmeyer-Peppas, and Hixon and Crowell). The release model that best fitted to the experimental data was selected according to the calculations of the coefficient of determination (R^2).

Model Name	Equation	F1	F2	F3	F4
**First Order model**	$\log Q_t = log\, Q_0 + \frac{k_i t}{2.303}$	0.85	0.75	0.37	0.14
**Higuchi model**	$Q_t = K_H \sqrt{t}$	0.88	0.90	0.58	0.76
**Korsmeyer-Peppas**	$\frac{Q_t}{Q_0} = K_k t^n$	0.93	0.95	0.82	0.90
**Hixon and Crowell**	$\sqrt[3]{Q_0} - \sqrt[3]{Q_t} = K_s t$	0.79	0.61	0.22	0.08

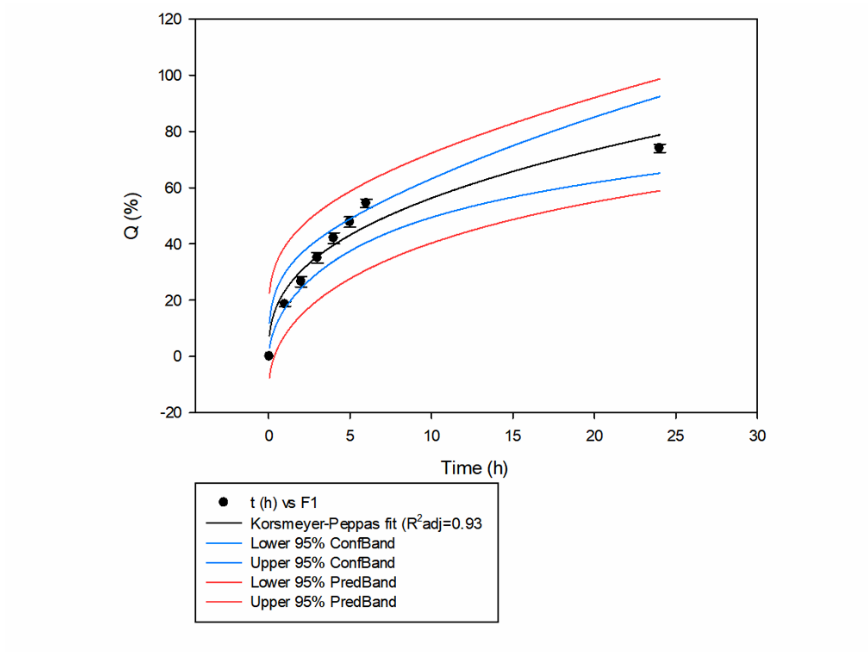

Figure S1. DXM release profile from F1. Continuous black line represents Korsmeyer-Peppas model fit. Blue lines correspond to upper and lower 95% confidence intervals and red lines upper and lower 95% prediction intervals.

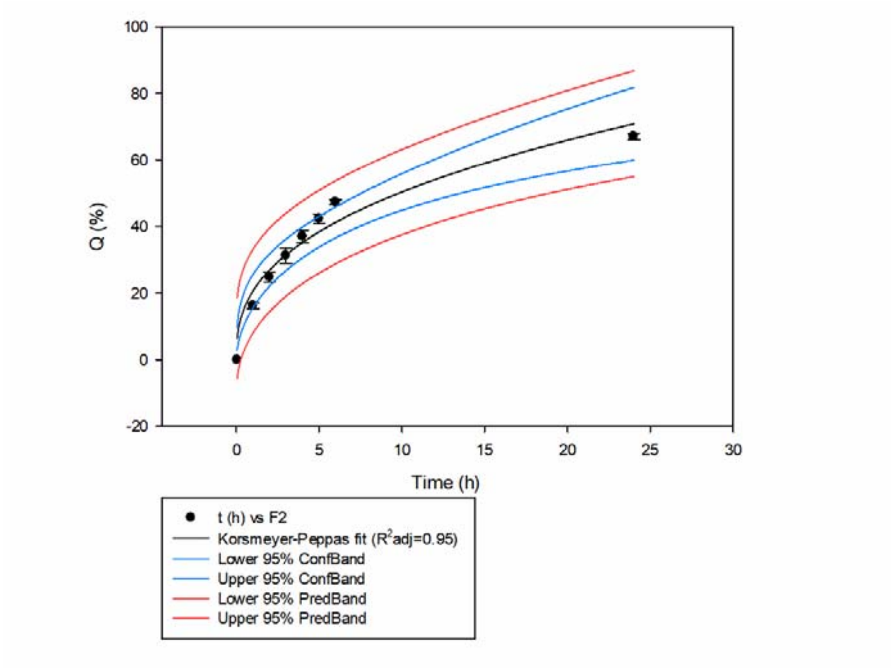

Figure S2. DXM release profile from F2. Continuous black line represents Korsmeyer-Peppas model fit. Blue lines correspond to upper and lower 95% confidence intervals and red lines upper and lower 95% prediction intervals.

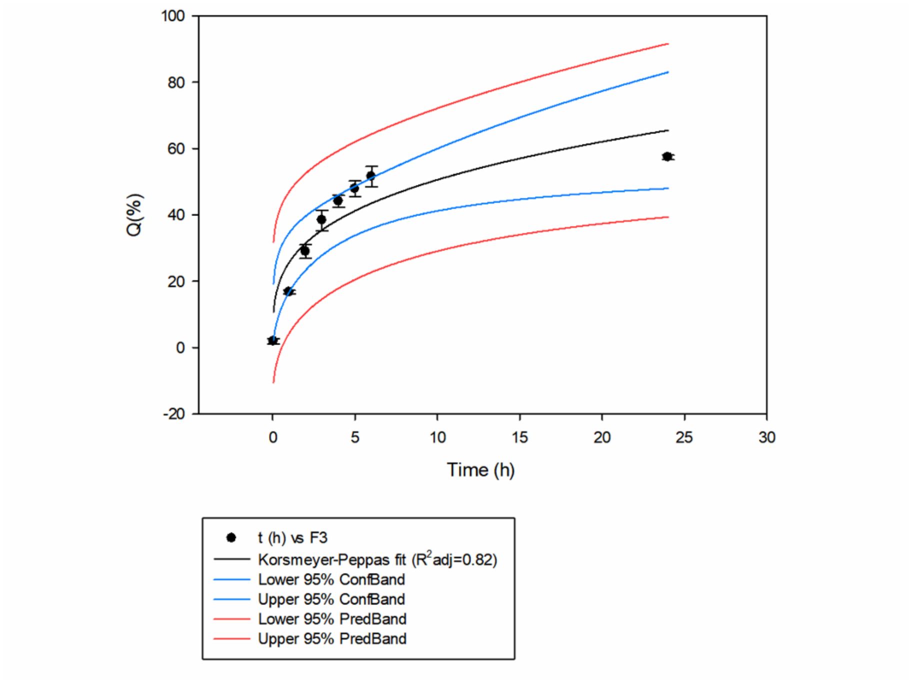

Figure S3. DXM release profile from F3. Continuous black line represents Korsmeyer-Peppas model fit. Blue lines correspond to upper and lower 95% confidence intervals and red lines upper and lower 95% prediction intervals.

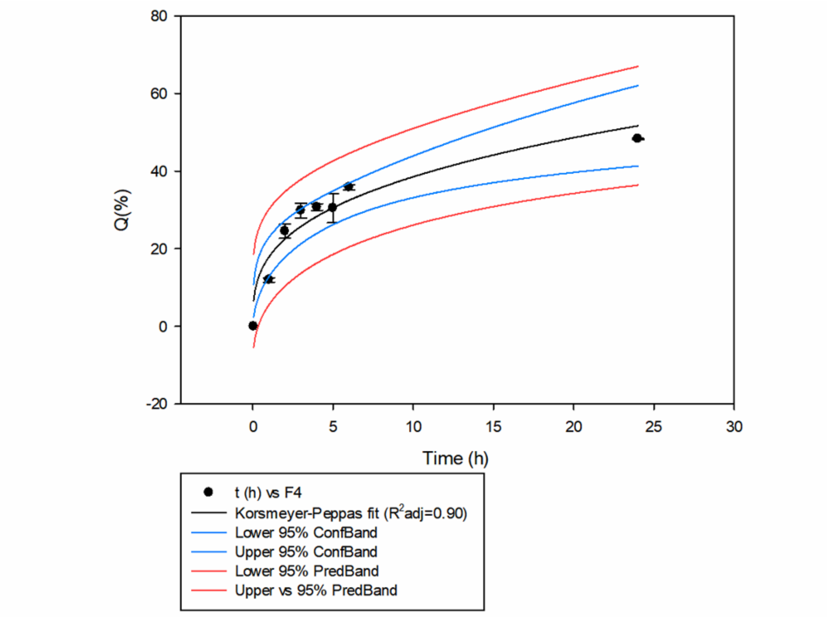

Figure S4. DXM release profile from F4. Continuous black line represents Korsmeyer-Peppas model fit. Blue lines correspond to upper and lower 95% confidence intervals and red lines upper and lower 95% prediction intervals.
